# Screening and identification of strains for high quality and antioxidant activity of *Baijiu* from strong-flavor *Daqu* and analysis of microbial synergistic effects

**DOI:** 10.1371/journal.pone.0319616

**Published:** 2025-03-10

**Authors:** Ruoyu Mi, Wei Lu, Xuan Zhang, Feng Yan, Beizhong Han, Qingyang Liu, Anjun Li, Ping Liu

**Affiliations:** 1 College of Food Science and Nutritional Engineering, China Agricultural University, Beijing, China; 2 Anhui Gujing Gongjiu Co. Ltd., Bozhou, China; 3 Department of Nutrition and Health, China Agricultural University, Beijing, China; Universidad San Francisco de Quito - Campus Cumbaya: Universidad San Francisco de Quito, ECUADOR

## Abstract

In this study, functional strains with strong fermentation characteristics were isolated from Strong-flavor *Daqu* and evaluated for their ability to enhance the quality and antioxidant activity of *Baijiu*. *Bacillus velezensis* (S1), *Bacillus subtilis* (S12), and *Escherichia coli* (S16) were identified as key strains. Fermentation experiments with different inoculation amounts and combinations revealed synergistic effects on *Baijiu* quality and antioxidant activity. Specifically, the total ester content in *Baijiu* fermented with 3% of S1, S12 and S16 were increased by 5.68%, 53.41% and 70.45% respectively, while the DPPH radical scavenging rate was increased by 52.01%, 17.63% and 35.52%, respectively, compared with the *Baijiu* fermented only with *Daqu*. Multi-strain combinations, particularly 4%-S16+2.5%-S1 and 3%-S16+4%-S12, exhibited notable antioxidant activity and ester content. Furthermore, the inoculation of 3% *E. coli*, 2% *B. velezensis* and 4% *B. subtilis* combination significantly increased total ester content (1.94 g/L) and antioxidant activity (38.09%) of *Baijiu*, in which S1 increased antioxidant activity of *Baijiu* while S12 increased total ester content. The results of GC-MS indicated that biofortified fermentation produced high levels of esters and guaiacol and its analogues, facilitated by synergistic interactions among indigenous microorganisms. This study is helpful to provide a new perspective and insight for improving *Baijiu* flavor and antioxidant activity.

## Introduction

Alcoholic liver disease (ALD), one of the most common liver diseases worldwide, is a series of liver injuries including steatosis, hepatitis, fibrosis, and cirrhosis caused by heavy alcohol consumption [1]. Oxidative stress injury caused by excessive ethanol intake [2] has been proven to be one of the pathogenic mechanisms of ALD, thus reducing oxidative stress is an effective strategy for preventing and treating ALD [3].

*Baijiu* is popular among consumers as a traditional Chinese fermented beverage. It has been found that moderate Strong-flavor *Baijiu* intake can attenuate mild ALD or non-alcoholic fatty liver disease (NAFLD) induced by ethanol intake in rats by through the glycerolipid pathway or the sirtuin-1/-adiponectin-dependent signal cascades [4,5]. Volatile compounds in *Baijiu*, including alcohols, esters, aldehydes, ketones, acids, acetals, furans, terpenes, nitrogenous compounds, and sulfurous compounds [6], which not only constitute the unique aroma and quality of *Baijiu*, but also exhibit a variety of bioactivities beneficial to health. Aromatic compounds, heterocyclic substances and esters in *Baijiu* have been found to be important in alleviating the oxidative stress on the liver induced by alcohol intake [7], and phenols contribute significantly to the antioxidant capacity of *Baijiu* [8]. 4-Methylguaiacol, 4-ethylguaiacol and vanillin in *Baijiu* have strong antioxidant activity in vitro analysis [9]. Zhao et al [10] found that 4-ethylguaiacol inhibits the activation of NF-κB and AP-1 by activating Nrf2-HO-1 and AMPK-SIRTI pathways, and reverses the inflammation induced by lipopolysaccharide. Thus, increasing the content of such antioxidant active compounds in *Baijiu* is the key to preventing ALD.

At present, studies have isolated microorganisms with specific function in *Daqu* and applied them to *Baijiu* fermentation to regulate the formation of volatile compounds and shorten the fermentation period, thereby improving the flavor and health quality of *Baijiu* [11,12]. Lactic acid bacteria (LAB) with good fermentation characteristics were isolated by multidimensional screening procedure and applied them to fermentation [13]. The results showed that the content of ethyl acetate, ethyl lactate, tetramethyl-pyrazine and 4-ethyl-2-methoxy-phenol in fermented liquor were significantly increased after inoculation with LAB. The fermentation process was enhanced with *Wickerhamomyces anomalus* with high yield of ethyl acetate isolated from *Daqu*, and as a result, the contents of ethyl acetate, ethyl caprate and phenyl acetate in *Baijiu* were increased [14]. High 3-methylthiopropanol-producing snap-capsule-coated yeasts were screened from sesame-flavor *Daqu* to confer floral, sweet, creamy, roasted nut, and clove-like aromas to *Baijiu* [15]. *Caldicoprobacter, Thermoactinomyces, Bacillus*, etc. contributes to the production of flavor substances or flavor precursors, which is important for improving the flavor of Strong-flavor *Baijiu* [16,17]. It is efficacious to screen out the strains with specific function to improve the quality of *Baijiu*. However, most of the studies on biofortification focus on the effect on improving the flavor of *Baijiu*, while there are few studies on the screening and application of strains with the function of regulating *Baijiu* health quality.

Microbial interactions influence community assembly, succession and stability and regulate food fermentation through nutrient exchange of metabolites [18]. However, the regulation of interactions between biofortification with the addition of functional strains and the microorganisms already present in the *Daqu* in *Baijiu* fermentation remains unclear. In this study, several strains were isolated and screened from Strong-flavor *Daqu* and characterized their performance in the fermentation process. At the same time, the quality and antioxidant activity of *Baijiu* were evaluated to screen out functional strains with good fermentation performance that can improve the antioxidant activity of *Baijiu*, and elucidated the effects of the inoculated amount and combination of these functional strains on the *Baijiu* quality. This study provides a theoretical basis for improving the flavor quality and antioxidant health quality of *Baijiu*.

## Materials and methods

### Materials

Glutinous sorghum was obtained from Liaoning province and the Strong-flavor *Daqu* was the product of Gujing Gongjiu Co., Ltd (Bozhou, China). The Yeast Extract Peptone Dextrose (YPD) medium, DeMan, Rogosa and Sharpe (MRS) medium and other reagents were all procured from Solarbio (Beijing, China).

### Isolation and screening of microorganisms in strong-flavor Daqu

Strong-flavor *Daqu* (10 g) was added to 90.00 mL of sterile 0.85% (w/v) NaCl and cultivated at 28 °C and 160 rpm for 30 min in a shaker (Haocheng HCY-DA, Suzhou, China) and then diluted for reserve. Serial dilutions (10^-2^, 10^-3^ until 10^-9^ fold) were prepared and 100 μL of each dilution was spread on YPD medium with 0.01% chloramphenicol, MRS medium with 0.20% natamycin, and Plate Count Agar (PCA) medium (5.0 g/L peptone, 2.5 g/L yeast extract, 1.0 g/L glucose, 15.0 g/L agar), which were used to culture yeasts, LAB and *Bacillus* species, respectively. Isolated colonies showed distinct morphologies were preserved with 40% (v/v) glycerol at - 80 °C. The conserved strains were incubated in YPD, MRS and PCA liquid medium at 28 °C and 160rpm for 24 h and the concentration of the culture was adjusted to 1×10^8^ Colony-Forming Units per Milliliter (CFU/mL) for further experiments.

### Starch degradation ability

The starch degradation ability was evaluated with sorghum solid medium (0.5% sorghum power, 0.1% peptone, 1.5% agar). The 10^-2^ fold gradient culture (1 μL) was spotted in the center of four equal portions of sorghum solid medium and then cultured at 30 °C for 48 h. Then add a few drops of dilute iodine solution to the medium. Iodine reacts with starch giving a dark blue substrate. Starch degradation ability was calculated using Eq 1:


R=Dh/Dc (1)

where *Dh* represents the clear zone diameter and *Dc* represents the colony diameter.

### α-Amylase and glucoamylase activity assays

Growth medium (YPD broth for yeast, MRS broth for LAB and PCA broth for bacillus) added with milled sorghum powder (2%), was inoculated with cell suspension (1%) of isolated strains and incubated at 30 °C for 5 days. The cultures were centrifuged at 8000 rpm for 10 min to obtain the supernatant, which served as crude enzyme solution. Evaluation of α-amylase and glucoamylase activities was carried out with α-amylase (AMS) assay kit and glucoamylase kit (Jiancheng Bioengineering institute, Nanjing, China), respectively, based on the manufacturer instructions.

### Determination of glucose utilization

The reducing sugar content (RSC) was determined by the 3,5-dinitrosalicylic acid (DNSA) following the method of Henry [19]. The stock solution of glucose was prepared by dissolving 10 mg of glucose in 1.00 mL of distilled water and diluted serially to be standard glucose solutions. The mixture of 1% Sodium hydroxide, 20% Rochelle salt, 0.05% Sodium sulfate, and 1% 3,5-Dinitro salicylic acid with 0.20 mL Phenol was kept at 95 °C for 5 min and then cooled to be DNS reagent. Then, take 300 μL of DNS reagent adding to 100 μL of standard glucose solutions and the crude enzyme solution, and the absorbance was measured at 540 nm and blanked with water for control.

### Determination of ethanol tolerance

To evaluate ethanol tolerance of strains, spot assay under the presence of ethanol were performed. Spot assay was performed by spotting 1 μL of 10-fold serial dilutions of the activated culture on ethanol-containing YPD, MRS, PCA medium. Ethanol concentrations were 0%, 4%, 8% and 10% (v/v), respectively. The plates were incubated at 30 °C for 48 h. Ethanol tolerance was calculated based on colony size.

### *Baijiu* fermentation

*Baijiu* was fermented by semi-solid state method in this research according to Liu et al. [20] with slight modifications. Crushed sorghum (125.0 g) was soaked in 60% distilled water (measured by weight of dry sorghum, 75.0 g) for 12 h and steamed for 1 h and then mixed with 30% Strong-flavor *Daqu* (measured by weight of dry sorghum, 37.5 g) and 240% distilled water (measured by weight of dry sorghum, 300.0 g). Then add the culture of different strains, respectively. All the fermentations were incubated for 17 days. Temperature setting for the fermentation process: warmed the mixture up to 33 °C and cooled it down to 28 °C slowly on the first 3 days, and then kept it until 17 days. The mixture fermented with isolated strains was the experimental groups, and without strains was used as the control (CK).

### Determination of total ester content

Total ester content was determined according to Complete Book of *Baijiu* Production Technology. 10.00 mL of *Baijiu* sample mixed with 2 drops of phenolphthalein indicator was titrated with a 0.1 mol/L NaOH standard solution until a pink color change was observed. The volume of NaOH standard solution utilized was recorded. Subsequently, 5.00 mL of 0.1 mol/L NaOH was added accurately, followed by the incorporation of zeolite and the installation of a condenser tube. The resulting mixture underwent reflux for 30 min in a boiling water bath. Upon cooling, titration with 0.1 mol/L HCl was conducted until the faint red color disappeared, signifying the endpoint. The volume of HCl standard solution consumed was then recorded.

### Antioxidant activities

#### DPPH free radical scavenging rate and hydroxyl radical scavenging rate.

The DPPH free radical scavenging rate (DPPH-RSA) is widely used in the evaluation of antioxidant ability [21]. DPPH-RSA determination was carried out according to the method of Zhang et al. [22] with slight modifications. The samples were diluted 2 times with sterile water and then the mixture of diluted sample and 0.13 mmol/L DPPH (Sigma, St. Louis, MO, USA) dissolved in 95% ethanol was shaken and maintained in the dark at 25 °C for 30 min. The absorbance of the solutions was measured at 515 nm (*A*_*1*_). Ethanol was used instead of the sample as a blank group (*A*_*0*_) and the control group substituted 95% ethanol for the DPPH solution (*A*_*2*_). The DPPH-RSA is calculated as Eq 2.


$$\%DPPH-RSA=\left(1-\frac{A_{1}-A_{2}}{A_{0}}\right)\times 100$$ (2)

The hydroxyl radical scavenging rate was measured according to the method of Zhang et al [23] with some modifications. 0.30 ml of 2.00 mg/mL FeSO_4_, 1.00 mL of 1% (w/v) H_2_O_2_ and 1.00 ml of 1.50 mg/mL salicylic acid alcoholic solution were added to the *Baijiu* sample respectively. Then the mixture was incubated at 37 °C water bath for 60 min and measured at 526 nm (*B*_*1*_). Substitute the salicylic acid alcoholic solution with distilled water and absolute ethanol to measure the absorbance value as blank (*B*_*0*_) and control group (*B*_*2*_). The Hydroxyl radical scavenging rate is calculated as Eq 3.


$$\%Hydroxylradicalscavengingrate\left(\text{\%}\right)=\left(1-\frac{B_{1}-B_{2}}{B_{0}}\right)\times 100$$ (3)

#### Total reducing power.

The total reducing power was measured according to the method of Zhang et al. [22] with modifications. 0.50 mL of *Baijiu* sample was added into 1.25 mL of sodium phosphate buffer (0.2 mol/L, pH 6.6) and 1.25 mL of K_3_Fe (CN)_6_ (0.01 g/mL) and the mixture was incubated in a water bath at 50 °C for 20 min. Subsequently, 1.25 mL of trichloroacetic acid (0.10 g/mL) was added and centrifuged for 10 min to take 1.50 mL supernatant. Then 1.50 mL of distilled water and 0.30 mL of ferric trichloride (0.001 g/mL) were added to the supernatant and was left to stand for 10 min. The absorbance of was determined at 700 nm as *C*_*1*_. In the blank group, distilled water was used instead of ferric trichloride (*C*_*0*_). The total reducing power is calculated as Eq 4.


$$Totalreducingpower=C_{1}-C_{0}$$ (4)

### Molecular biological identification

The technically well characterized strains were selected for molecular biology identification. The DNA was extracted by a DNA extraction kit (TSINGKE TSP101, Beijing, China). 16S rDNA and ITS were amplified using universal primers (27F/1492R and ITS1/ITS4) respectively. Polymerase chain reaction (PCR) amplification products were detected by 1% agarose gel electrophoresis, and sent to Tsingke Biotech Co., Ltd. (Beijing, China) for sequencing. This sequence was specifically compared to the GenBank database using NCBI BLAST and the standard microorganisms with similar genetic relationships were analyzed to construct a phylogenetic tree.

### GC-MS analysis of volatile compounds

*Baijiu* diluted 5 times was placed in a 20.00 mL headspace vial, added 2 g of NaCl and 200.00 μL of the 4-methyl-2-pentanol (1.0 mg/mL). Volatile compounds were collected in a SPME fiber (DVB/CAR/PDMS, 50/30 μm, Supelco Co., Bellefonte, PA, USA) at 50 °C for 45 min. Then the fiber was inserted into the GC injection port at 250 °C for 5 min, analyzed with GC-MS (Agilent Technologies 7890A-5975C, Palo Alto, CA, USA) subsequently. The chromatographic conditions were as follows: chromatographic column (DB-WAX, 30 m × 0.25 mm × 0.25 μm, Agilent, USA); injection inlet temperature: 240 °C, programmed temperature: 40 °C for 3 min, then increased to 240 °C at a rate of 3 °C/min, holding 10 min; split ratio, 10:1; carrier gas, helium (99.999%). Mass spectrometry conditions were as follows: Interface temperature: 230 °C; ion source temperature 230 °C; ionization: EI, electron energy: 70 eV; the scan mass range m/z: 20-550. 4-methyl-2-pentanol (1.0 mg/mL) was used as the internal standard to determine semi-quantitatively the content of the volatile compounds.

### Data management and statistical analysis

The volatile compounds were putatively identified by MS spectra matching using National Institute of Standards and Technology (NIST14.L) spectral database. Compounds with matching degree less than 70% were selectively removed from the results. The volatile compounds data were analyzed to create a heat map by Min-Max normalization according to Eq 5:


$$X^{*}=\frac{X-X_{min}}{X_{max}-X_{min}}$$ (5)

where X* is the data after normalization; *X* is the raw data; *X*_*min*_ is the minimum value of data; *X*_*max*_ is the maximum value of the data.

Normality and homogeneity of variance of experimental data were tested. Non-parametric tests are conducted on data that do not satisfy the conditions. For the data conformed normality and homogeneity of variance, statistical comparisons among means were conducted using One-Way Analysis of Variance (ANOVA) with Duncan’s post hoc test with SPSS 22.0 software.

## Results and discussion

### Technological characterization of isolated strains

#### Starch degradability and the activity of α-amylase and glucose amylase.

Sixteen strains, showed significant differences in the morphology of colonies and accounted for a large proportion in the microbial community of *Daqu* were initially screened from Strong-flavor *Daqu*, numbered S1, S2, ... and S16. The 16 strains were incubated at 30°C for 48 h on the sorghum solid medium. The strains which could hydrolyze starch displayed transparent clear zones around their colonies after adding a few drops of dilute iodine solution to the medium, while those lacking this ability did not produce such zones. The ratio of the clear zone diameter (Dh) to colony diameter (Dc) was calculated to evaluate the starch degradation capacity of strains. The study revealed that about 9 strains exhibited starch degrading power, with strain S1 demonstrating the strongest ability to degrade starch (Table 1). Ten strains were able to produce α-amylase and S16 exhibited the highest α-amylase producing activity (95.33 U/L), followed by S1 (81.04 U/L). S1, S15, and S16 exhibited a high level of glucose amylase production capacity and they could be used as intensive fermentation microorganisms to enhance starch utilization and ethanol production in *Baijiu* fermentation [24].

**Table 1:** The fermentation characteristics of all strains.

Strain Number	R (Dh/Dc)	α-Amylase activity (U/L)	Glucose amylase activity (U/L)	Glucose utilization (%)
**S1**	1.34 ± 0.07	81.04 ± 2.71	26.38 ± 1.34^b^	31.28 ± 0.63^c^
**S2**	1.09 ± 0.02	46.79 ± 1.54	21.71 ± 1.23 cd	28.56 ± 0.92^d^
**S3**	0.00 ± 0.00	58.52 ± 2.87	4.78 ± 0.87^l^	6.53 ± 1.63^g^
**S4**	0.00 ± 0.00	0.00 ± 0.00	15.34 ± 0.92^gh^	96.56 ± 0.71^a^
**S5**	1.21 ± 0.06	37.13 ± 4.03	17.42 ± 1.46^f^	23.78 ± 1.62^f^
**S6**	1.18 ± 0.08	25.38 ± 1.42	20.07 ± 1.23^de^	25.24 ± 1.34^ef^
**S7**	0.00 ± 0.00	0.00 ± 0.00	13.86 ± 1.29^hi^	96.56 ± 0.23^a^
**S8**	0.00 ± 0.00	0.00 ± 0.00	8.12 ± 1.47^k^	96.66 ± 0.16^a^
**S9**	1.03 ± 0.05	12.58 ± 1.67	11.42 ± 1.07^j^	26.18 ± 0.68^e^
**S10**	0.00 ± 0.00	63.48 ± 4.03	3.10 ± 0.27^l^	5.7 ± 1.72^g^
**S11**	1.23 ± 0.05	35.78 ± 1.74	15.52 ± 1.18^gh^	6.37 ± 1.16^g^
**S12**	1.25 ± 0.00	37.21 ± 1.57	19.41 ± 1.42^e^	96.31 ± 0.18^a^
**S13**	0.00 ± 0.00	0.00 ± 0.00	12.56 ± 0.37^ij^	96.48 ± 0.81^a^
**S14**	0.00 ± 0.00	0.00 ± 0.00	16.52 ± 0.83^fg^	96.55 ± 0.06^a^
**S15**	1.27 ± 0.05	0.00 ± 0.00	22.35 ± 0.54^c^	96.63 ± 0.37^a^
**S16**	1.31 ± 0.08	95.33 ± 1.52	28.31 ± 0.78^a^	36.28 ± 0.48^b^

The data of R and α-Amylase activity uses non-parametric tests. Different lowercase letters indicate differences between each group (p <  0.05).

#### Glucose utilization.

In the process of *Baijiu* fermentation, microorganisms and enzymes in fermented grain collaborate to hydrolyze starch to accessible nutrients, which are subsequently transformed by microorganisms into diverse flavor compounds to enrich the quality of *Baijiu* [25–29]. The results showed that all 16 strains consumed glucose for growth (Table 1). Seven strains demonstrated glucose utilization above 96%, indicating their promise as functional strains for *Baijiu* fermentation.

#### Ethanol tolerance.

During the fermentation process, yeasts produce ethanol through alcoholic fermentation. Ethanol not only contributes to the flavor of *Baijiu* but also acts as a precursor for the synthesis of other important compounds, such as esters and acids [30–32]. The presence of high ethanol concentrations during *Baijiu* fermentation inhibits microbial growth, thereby impacting the quality of *Baijiu* [33]. All strains exhibited robust growth within an alcohol concentration range of 4%. Four strains (S1, S9, S12, S15) showed growth tolerances on 10% (v/v) ethanol, and S2, S4, S5 and S6 were able to tolerate up to 8% (v/v) ethanol. The impact of ethanol on strain growth varied significantly, with ethanol tolerance differing markedly among strains, which may be caused by the type of strain or the individual performance of the strain [34].

### Effects of inoculated S1, S12 and S16 on total ester content and antioxidant activity of *Baijiu*

#### Total ester content.

**Fig 1:**
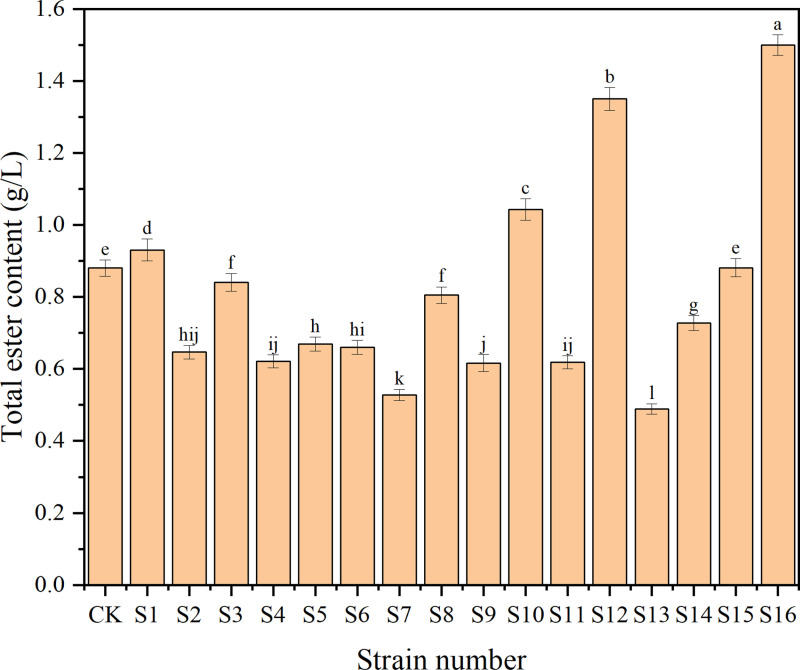
Total ester content of *Baijiu* fermented by isolated strains. The alphabet above the chart means the significant difference (p <  0.05).

The culture of the 16 strains (3% of sorghum, w/w) was inoculated into a mixture of treated sorghum and *Daqu* (30% of sorghum, w/w) for laboratory-scale semi-solid fermentation. Total ester is an important physicochemical index in the analysis of *Baijiu*, reflecting the type and content of aroma substances which is an important indicator for assessing the quality of *Baijiu* [6]. As shown in Fig 1, five groups of strains fermented *Baijiu* with higher total ester content than the control group (CK), which has the potential to improve the quality of *Baijiu* (p <  0.05). The group S16 exhibited the greatest increase in total esters, with a 70.45% rise compared to CK while the group S12 increased by 53.41% compared to CK.

#### Antioxidant activity.

**Fig 2:**
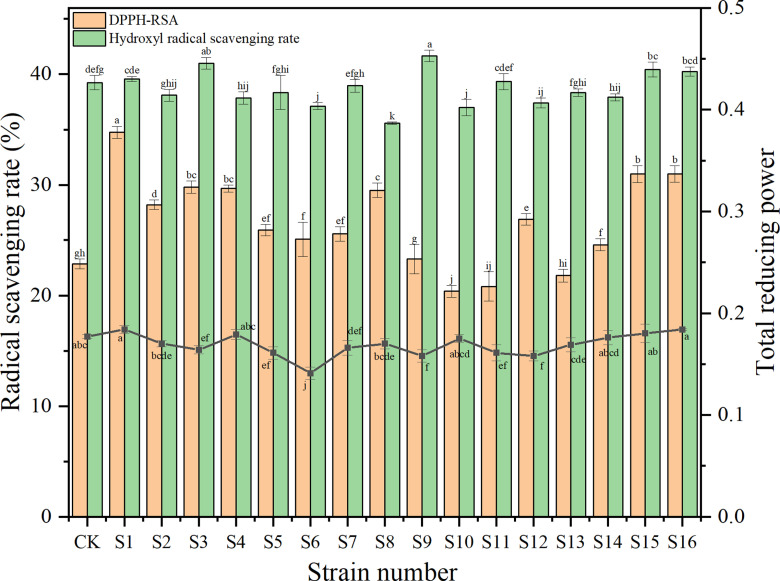
The antioxidant activities of *Baijiu* fermented by isolated strains. The alphabet above the chart means the significant difference (p <  0.05).

DPPH-RSA and total reducing power were used as evaluation indexes to assess the antioxidant activity of brewed *Baijiu* [22]. In this study, the above indices were determined in fermented *Baijiu* inoculated with 16 functional strains to evaluate the antioxidant activity (Fig 2). The DPPH-RSA of *Baijiu* were higher in 13 groups compared to CK (p <  0.05). S1 exhibited the highest improvement, with a 52.01% increase compared to CK, followed by S15 and S16 with a 35.52% increase. However, DPPH-RSA was decreased in S10, S11 and S13 compared to CK. The scavenging rate of hydroxyl radical in S9 was the highest (41.66%), but only 6.17% higher than that in CK. The total reducing power of Baijiu was in the range from 0.141-0.184, with S16 showing the highest total reducing power and S6 the lowest.

In summary, in combination with the total ester content, *Baijiu* fermented with S3 showed inconsistencies in DPPH-RSA and reducing power results, which could not be fully demonstrated to have the potential of enhancing antioxidant activity. Although S15 improved the antioxidant activity of *Baijiu*, the total ester was not significantly increased. S1 slightly increased the total ester content but significantly increased the antioxidant activity, while S12 showed the opposite and S16 revealed higher total ester and antioxidant activity at the same time. To study the synergies between strains, these three strains were further characterized and effects of the double and triple combination of strains on the total ester and antioxidant activity of *Baijiu* were studied.

### Strain S1, S12 and S16 identification

**Fig 3:**
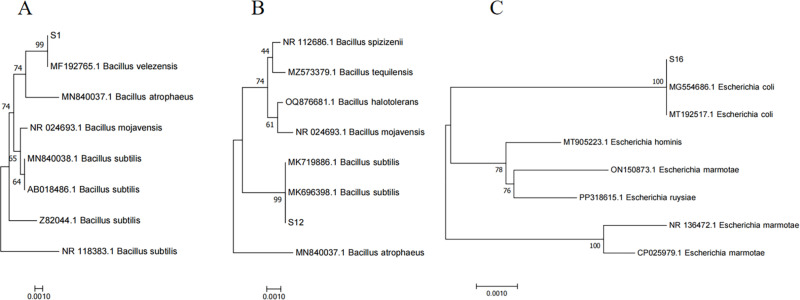
Neighbor-joining tree based on 16S rRNA gene sequences showing the phylogenetic relationship. (A) S1; (B) S12; (C) S16.

It was observed that the colony morphology of S1 and S12 was opalescent and grayish-white, with folded surface and irregular edges. And the colony morphology of S16 was opaque and opalescent with a smooth surface. Sequence comparisons were carried out using the MEGA-X method, and the phylogenetic tree was constructed by using the Neighbor-Joining method of Multiple Sequence Alignment (MSA) analysis. As shown in Fig 3, S1, S12, and S16 belong to the same branch with *Bacillus velezensis*, *Bacillus subtilis*, and *Escherichia coli*, respectively. The three strains can be identified combined with the colony morphology analysis.

### Effect of inoculated amount of isolated strain S1, S12 and S16 on total ester content and antioxidant activity of *Baijiu*

Based on the above results, strains S1, S12 and S16, which were able to ferment high quality and antioxidant *Baijiu*, were inoculated with 1%-4% strain solution, respectively, and the effects of different inoculation amounts of strain solution fermented with glutinous sorghum and 30% (w/w) *Daqu* on *Baijiu* quality and antioxidant activity were studied.

**Fig 4:**
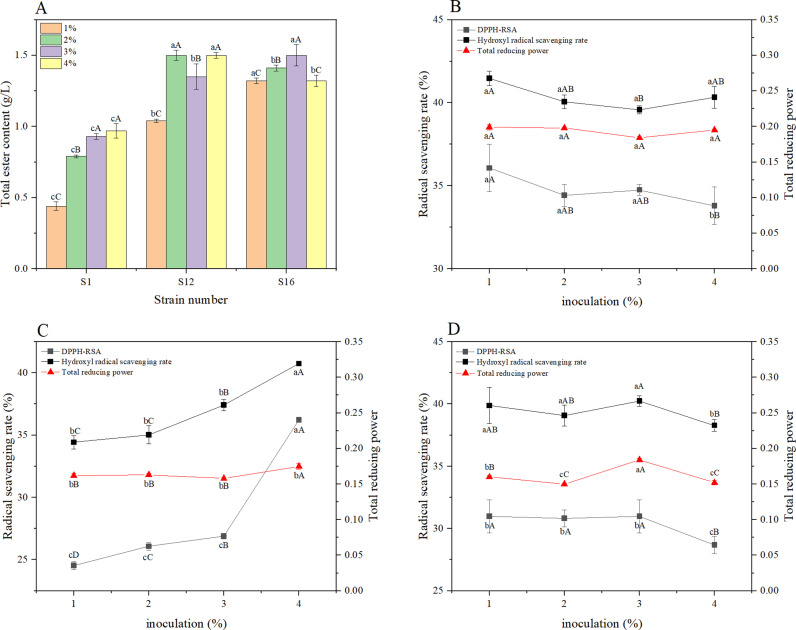
The total ester content and antioxidant activities of *Baijiu* fermented by S1, S12 and S16. (A) total ester content; (B) antioxidant activities of *Baijiu* fermented by S1; (C) antioxidant activities of *Baijiu* fermented by S12; (D) antioxidant activities of *Baijiu* fermented by S16. The alphabet above the chart means the significant difference (p <  0.05). Lowercase letters indicate differences between groups, and uppercase letters indicate differences within groups.

As shown in Fig 4, compared with CK (fermented without strain solution), the addition of 1%-2% S1 decreased the total ester of Baijiu slightly, and 4% S1 increased the total ester of liquor by 10.23%. However, the addition of S1 significantly increased the DPPH-RSA and the total reducing power of *Baijiu*. The DPPH-RSA, hydroxyl radical scavenging rate and total reducing power of 1% S1 fermented *Baijiu* were increased by 57.79%, 5.68% and 12.43%, respectively. The total ester content of fermented *Baijiu* with different inoculated amount of S12 was significantly higher, increased by 18.18%-70.45%, the DPPH-RSA was slightly increased, and the total reducing power was lower than that of CK. *Bacillus* species are an important microorganism in fermentation, which have been found to enhance the antioxidant activity of some fermented foods. Previous studies have shown that adding *B. subtilis* during tamarind seed fermentation can increase the DPPH-RSA and Ferric ion reducing antioxidant power of product [35]. During *Baijiu* fermentation, *Bacillus* produces tetramethylpyrazine and other substances with antioxidant activity, thus improve the antioxidant activity of *Baijiu* [36,37], which is consistent with our findings. The total ester content increased gradually with the increase of S16 inoculation, and the antioxidant activity of *Baijiu* was elevated when the amount was added up to 3%, which increased the DPPH-RSA, hydroxyl radical scavenging rate and total reducing power by 35.52%, 2.57% and 3.95%, and the total ester by 70.45% compared with CK, respectively.

### Effects of strains synergies on total ester and antioxidant activity of *Baijiu*

#### Double strains combination.

According to the results of fermentation with single strain, the total ester and antioxidant activities of *Baijiu* were good for different inoculums of S16, while S1 and S12 both had some limitations so that S16 was compounded with S1, and S12, respectively, to study the effect of adding two functional strains on the fermentation of *Baijiu*.

**Fig 5:**
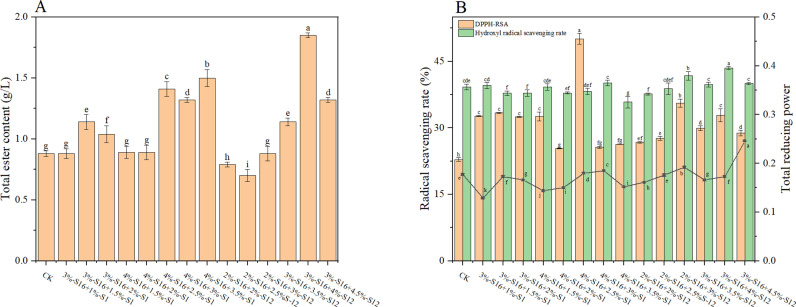
The total ester content and antioxidant activities of *Baijiu* fermented by double strains. (A) total ester content; (B) antioxidant activity. The alphabet above the chart means the significant difference (p <  0.05).

As shown in Fig 5, the total ester content of the S16 + S12 combination system increased, but too high or too low inoculated amounts of strains affected the total ester content and antioxidant activity. The quality of *Baijiu* inoculated with 3%-S16 + 4%-S12 was significantly improved, and the total ester content was increased by 110.23% compared to CK. But the antioxidant activity index DPPH-RSA and total reducing power were lower than that of single strain, which might be due to microbes competing for resources and inhibiting their effects [38].

3%–4% of S16 was combined with 1%–3.5% of S1, and it was found that the overall antioxidant activity of S16 + S1 combination system was higher than that of single strain, but the total ester content was relatively lower than that of single S16. The DPPH-RSA of 4%-S16 + 2.5%-S1 fermented *Baijiu* was 50.11%, which was 119.20% higher than that of CK and 61.75% higher than that of single S16.

#### Three strains combination.

**Fig 6:**
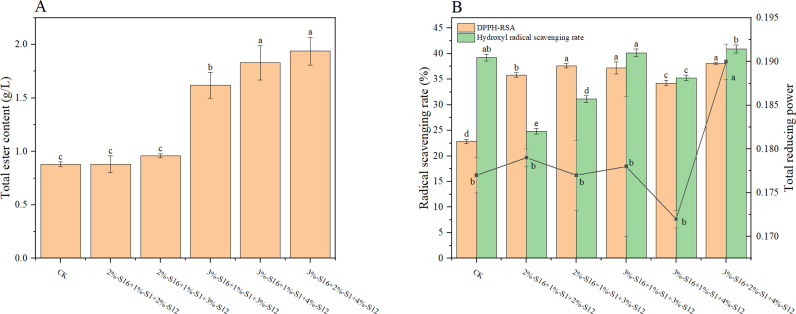
The total ester content and antioxidant activities of *Baijiu* fermented by three strains. (A) total ester content; (B) antioxidant activity. The alphabet above the chart means the significant difference (p <  0.05).

According to the results of the combination of double strains, adding appropriate concentration of S12 could increase the total ester content of *Baijiu*, and adding strain S1 could increase the DPPH-RSA. Therefore, the quality and antioxidant activity of *Baijiu* inoculated with different concentrations of three strains were further studied. The quality and antioxidant activity of 3%-S12 + 2%-S1 + 4%-S12 were the best, and the total ester content was 1.94 g/L (Fig 6), which was higher than that of single and double strains combined *Baijiu*. The scavenging rate of DPPH free radical was 38.09%, which was higher than that of single S16 and S12 but lower than that of 4%-S16 + 2.5%-S1, which was possibly because S12 inhibited the effect of S1 at this concentration. The combination of *B. subtilis* and *B. velezensis* has been used in previous study of fortified *Daqu*, and the results showed that the total contents of esters and phenols in fermented *Baijiu* with fortified *Daqu* have significantly increased [39].

### Effects of selected strains on volatile compounds of fermented *Baijiu*

**Fig 7:**
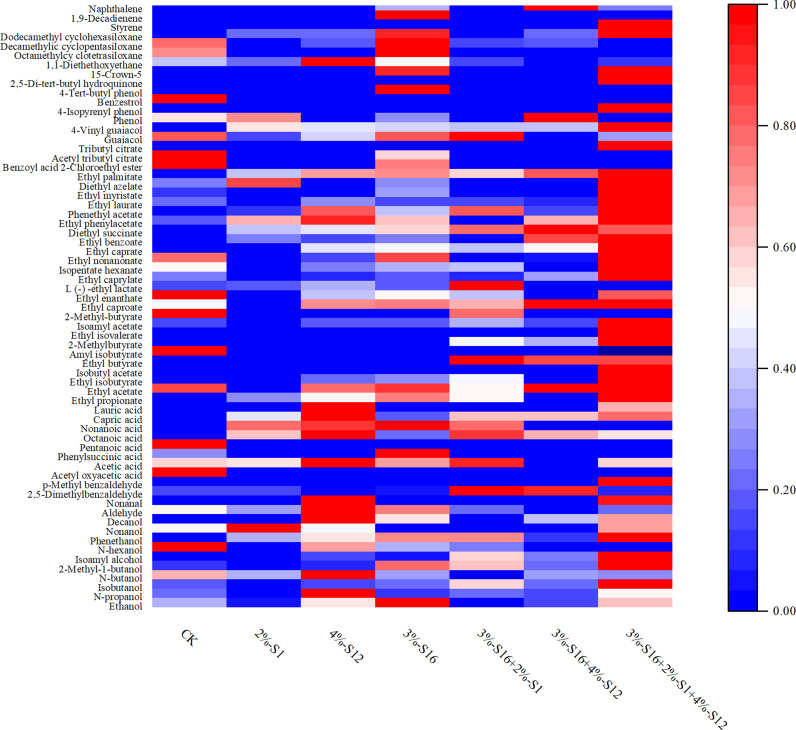
The heatmap of volatile compounds in *Baijiu* fermented with different strain combination. The data in the heatmap is analyzed by Min-Max normalization.

Flavor compounds are important characteristic which reflect the basic quality of *Baijiu*, and the interaction of different flavor molecules provides *Baijiu* with a unique flavor. The aim of this part was to investigate the effects of the isolated *B. velezensis*, *B. subtilis* and *E. coli* and their combinations on flavor metabolites during *Baijiu* fermentation. Totally, 65 flavor compounds were detected, including 10 alcohols, 4 aldehydes, 8 acids, 28 esters, 7 phenols and 8 other compounds using GC-MS as shown in Fig 7. Differences in the content of flavor compounds among the treatment groups were attributed to changes in the structure of the microbial community in the fermentation system resulted from the addition of the fortified strains, which in turn was attributed to the metabolic effects of the altered bacterial flora and to the chemical reactions that occur between the different flavor compounds.

Esters are the most varied of all flavor compounds and determine the flavor, aroma, grade and style of *Baijiu* [24,40,41]. As shown in Fig 7, the ester content of 3%-S16 + 2%-S1 + 4%-S12 was the highest (905.31 mg/kg), and the ester content of *Baijiu* fermented with *B. subtilis* was significantly higher than that of others, which was consistent with the results of a previous study [42]. Ethyl caproate is the characteristic aroma of Strong-flavor *Baijiu* [11], which has a fruity flavor. The content of ethyl caproate was 10.21% and 10.47% higher in *Baijiu* fermented with 4% *B. subtilis* and 3% *E. coli* alone than that in CK group, and was increased by 21.23% in 3%-S16 + 2%-S1 + 4%-S12, respectively. The contents of ethyl acetate, ethyl caprylate, ethyl caprate and ethyl palmitate were prominent in the *Baijiu*, contributing fruity, coconut and creamy flavor. Double and three bacterial combinations fermented *Baijiu* with higher content of these substances than single strain. Ethyl palmitate increases the mellowness of *Baijiu*, reduces its dryness, enhances the aftertaste, and plays a crucial role in the stability and taste [43,44]. In addition, isoamyl acetate (strong fruity and floral aroma), ethyl butyrate (pineapple and banana odor), ethyl benzoate (fruity aroma), phenethyl acetate (honey aroma), and ethyl laurate (peanut aroma) contribute to the special aroma of *Baijiu* [31,45].

Ten alcohols were detected in the samples, with high percentages of ethanol, isoamyl alcohol and phenylethyl alcohol. Ethanol is the most dominant substance in *Baijiu* and determines the yield. The ethanol content of all experimental groups was higher than CK except 2%-S1, and the highest ethanol content was found in the 3%-S16 group (957.21 mg/kg). *Saccharomyces cerevisiae* is effective for ethanol production [46], and the addition of *B. subtilis* modulated the structure of the microbial community in the spirits and assisted the *S. cerevisiae* to play its role. Acids not only provide a special flavor for *Baijiu* but also are a prerequisite for the synthesis of esters, such as lactic acid which provides sour aroma and eliminates the bitter and pungent taste of *Baijiu* [47]. Addition of *Bacillus* resulted in an increase in the synthesis of octanoic acid, the precursor of ethyl decanoate, thus promoting the formation of ethyl decanoate [14]. The acid content of all experimental groups was higher than that of CK, while the aldehyde content decreased.

Phenols are key aromatic compounds in *Baijiu* and have antioxidant activities [17] especially guaiacol and its analogues, 4-methyl guaiacol, 4-ethyl guaiacol and 4-vinyl guaiacol. The content of guaiacol in *Baijiu* fermented with only *Daqu* was 1.45 mg/kg and 4-vinyl guaiacol was not detected, while the contents of 4-vinyl guaiacol were higher than 17.00 mg/kg in experimental groups *Baijiu* with enhanced strains. In particular, the content of 4-vinyl guaiacol in 3%-S16+2%-S1+4%-S12 group was 42.54 mg/kg. Previous studies have shown that some Bacillus are associated with the production of phenols. In the fermentation of sesame-flavored *Baijiu*, *B. licheniformis* was positively associated with guaiacol, and 4-vinyl guaiacol [48]. The addition of *B. subtilis* increased the content of phenolic substances in Chinese yellow rice wine (Huangjiu) [49]. Similar results proved that the addition of *Bacillus* could improve the antioxidant activity of liquor, which was consistent with the DPPH-RSA results.

## Conclusion

In this study, 16 strains were isolated from *Daqu* and 3 functional strains were identified based on technological characterization and antioxidant activity of fermented *Baijiu*. The combined fermentation of these strains was investigated for its impact on *Baijiu* quality, antioxidant activity, and flavor compounds. Results demonstrated enhanced quality and antioxidant activity of *Baijiu* through bioaugmentation fermentation with *B. velezensis*, *B. subtilis*, and *E. coli*. Combined with the results of GC-MS, it was found that *E. coli* had good ability to produce ester and antioxidant active substances at the same time. *B. velezensis* increased the antioxidant activity of *Baijiu* and the DPPH-RSA of 4%-S16+2.5%-S1 fermented *Baijiu* increased by 119.20%. While *B. subtilis* increased the content of esters during fermentation and the total ester content of 3%-S16+4%-S12 significantly increased by 110.23%. The total ester and antioxidant activity of *Baijiu* fermented with 3%-S16+2%-S1+4%-S12 were the best. Varied addition amounts and combinations of enhanced fermented *Baijiu* showed different characteristics, indicating that it had different effects on the structure of microbial community.

This study completed the screening and identification of enhanced microorganisms and the effect on *Baijiu* quality, and provide a reference for improving the functional quality of *Baijiu* by bioaugmentation. Future studies should focus on elucidating the effects of enhanced strains on fermentation system, including the dynamic changes of physicochemical indexes and microbial community structure of fermented grains for further interpretation.

## Supporting information

S1 Table Volatile compounds in *Baijiu* fermented with different strain combination. (DOCX)
