# Supplementary material for: Screening and identification of strains for high quality and antioxidant activity of Baijiu from strong-flavor Daqu and analysis of microbial synergistic effects
Source: PLoS One. 2025 Mar 10;20(3):e0319616. doi: 10.1371/journal.pone.0319616 (PMC11892878; doi:10.1371/journal.pone.0319616)
Supplement: S1 Table — (DOCX) [file pone.0319616.s001.docx]

**S1 Table. Volatile compounds in *Baijiu* fermented with different strain combination.**

| Compound (mg/kg) | CK | 2%-S1 | 4%-S12 | 3%-S16 | 3%-S16+2%-S1 | 3%-S16+4%-S12 | 3%-S16+2%-S1+4%-S12 |
| --- | --- | --- | --- | --- | --- | --- | --- |
| Ethanol | 872.33±42.31 | 834.19±25.88 | 896.80±2.84 | 957.21±48.68 | 826.83±13.37 | 845.96±2.98 | 907.37±10.32 |
| N-propanol | 2.89±0.14 | 2.63±0.21 | 3.85±0.01 | 2.78±0.13 | 2.91±0.08 | 2.82±0.04 | 3.27±0.17 |
| Isobutanol | 24.59±1.19 | 19.44±1.36 | 24.02±0.09 | 25.80±0.66 | 37.29±1.29 | 25.53±0.34 | 49.20±2.43 |
| N-butanol | 0.81±0.04 | 0.66±0.04 | 1.00±0.03 | 0.63±0.08 | 0.47±0.03 | 0.64±0.03 | 0.62±0.03 |
| 2-Methyl-1-butanol | 55.54±2.69 | 45.54±1.59 | 51.54±0.71 | 106.96±2.07 | 93.57±2.03 | 62.24±0.52 | 122.51±0.35 |
| Isoamyl alcohol | 141.82±6.88 | 140.73±11.57 | 162.11±1.14 | 148.20±0.66 | 221.40±8.80 | 173.13±1.07 | 279.25±2.65 |
| N-hexanol | 3.24±0.16 | -- | 2.19±0.00 | 1.12±0.06 | 0.85±0.00 | -- | -- |
| Phenethanol | 46.17±2.24 | 78.34±3.57 | 98.03±8.71 | 114.13±10.90 | 113.03±4.04 | 57.86±1.43 | 141.39±1.44 |
| Nonanol | 2.27±0.11 | 4.52±0.20 | 2.23±0.03 | -- | -- | -- | 3.08±0.45 |
| Decanol | -- | -- | 7.40±0.09 | 4.05±0.14 | -- | 2.89±0.04 | 4.94±0.37 |
| Aldehyde | 4.66±0.23 | 3.73±0.22 | 6.82±0.02 | 5.73±0.11 | 3.32±0.13 | 2.35±0.06 | 3.34±0.08 |
| Nonanal | -- | -- | 1.60±0.31 | -- | -- | -- | 1.50±0.22 |
| 2,5-Dimethylbenzaldehyde | 17.37±0.84 | 17.21±1.05 | 10.27±0.38 | 12.72±1.82 | 57.89±3.06 | 54.71±1.05 | 14.66±0.87 |
| p-Methyl benzaldehyde | -- | -- | -- | -- | -- | -- | 3.30±0.07 |
| Acetyl oxyacetic acid | 1.92±0.09 | -- | -- | -- | -- | -- | -- |
| Acetic acid | 0.80±0.04 | 0.73±0.01 | 1.35±0.08 | 0.92±0.00 | 1.24±0.07 | -- | 0.79±0.03 |
| Phenylsuccinic acid | 1.21±0.06 | -- | -- | 4.11±0.01 | -- | -- | -- |
| Pentanoic acid | 1.05±0.05 | -- | -- | -- | -- | -- | -- |
| Octanoic acid | 2.30±0.11 | 6.13±0.22 | 8.66±0.06 | 3.61±0.11 | 8.00±0.78 | 6.48±0.35 | 5.87±0.76 |
| Nonanoic acid | -- | 2.08±0.10 | 2.29±0.08 | 2.63±0.26 | 2.05±0.58 | -- | -- |
| Capric acid | 3.07±0.15 | 9.38±2.37 | 16.81±0.30 | 5.56±0.02 | 11.59±4.30 | 11.33±0.40 | 13.71±0.78 |
| Lauric acid | -- | -- | 3.83±0.60 | -- | -- | -- | 2.53±0.70 |
| Ethyl propionate | -- | 0.28±0.03 | 0.49±0.03 | 0.74± | 0.52±0.04 | -- | 0.98±0.04 |
| Ethyl acetate | 83.36±1.42 | 35.87±2.88 | 79.95±0.99 | 84.50±3.22 | 63.55±2.07 | 89.88±3.15 | 91.17±2.46 |
| Ethyl isobutyrate | -- | -- | 0.39±0.03 | 0.51±0.30 | 0.88±0.04 | -- | 1.86±0.01 |
| Isobutyl acetate | -- | -- | -- | -- | -- | -- | 2.14±0.03 |
| Ethyl butyrate | -- | -- | -- | -- | 2.07±0.66 | 1.74±0.11 | 1.76±0.02 |
| Amyl isobutyrate | 1.09±0.02 | -- | -- | -- | -- | -- | -- |
| 2-Methylbutyrate | -- | -- | -- | -- | 0.73±0.11 | 0.55±0.02 | 1.51±0.02 |
| Ethyl isovalerate | -- | -- | -- | -- | -- | -- | 0.88±0.06 |
| Isoamyl acetate | 9.24±0.75 | 6.77±0.46 | 10.05±0.01 | 9.98±3.10 | 12.60±5.63 | 9.48±0.05 | 23.51±0.11 |
| 2-Methyl-butyrate | 0.95±0.04 | -- | -- | -- | 0.73±0.11 | -- | -- |
| Ethyl caproate | 23.50±1.24 | 18.44±1.08 | 25.90±0.45 | 25.96±0.07 | 25.06±1.64 | 28.66±0.78 | 28.49±0.18 |
| Ethyl enanthate | 4.03±0.67 | -- | 1.59±0.07 | 2.03±0.67 | 1.50±0.11 | -- | 3.35±0.04 |
| L (-) -ethyl lactate | 2.23±0.43 | 3.01±0.11 | 5.52±0.44 | 3.05±0.14 | 15.90±0.43 | -- | -- |
| Ethyl caprylate | 83.31±1.67 | 55.71±1.82 | 64.38±1.33 | 73.02±4.87 | 62.65±4.33 | 88.12±3.11 | 159.32±4.14 |
| Isopentate hexanate | 1.99±0.41 | -- | 1.03±0.02 | 1.44±0.11 | 1.59±0.66 | -- | 3.98±0.29 |
| Ethyl nonanonate | 11.18±0.56 | 3.54±0.13 | 4.89±1.41 | 12.10±1.80 | 3.58±1.09 | 4.02±0.32 | 13.42±0.54 |
| Ethyl caprate | 102.23±12.71 | 98.59±1.87 | 178.79±2.08 | 184.14±15.75 | 168.18±11.30 | 190.44±2.44 | 281.56±19.47 |
| Ethyl benzenecarboxylate | -- | 6.53±0.26 | 3.33±0.10 | 6.38±0.11 | -- | 21.21±0.21 | 24.92±1.47 |
| Diethyl succinate | 2.30±0.85 | 3.89±0.26 | 4.23±1.47 | 4.77±0.02 | 5.61±0.44 | 6.61±0.42 | 5.77±0.14 |
| Ethyl phenylacetate | 4.26±0.78 | 7.91±0.80 | 9.91±0.26 | 7.82±0.23 | 2.97±0.46 | 7.92±0.16 | 10.65±0.48 |
| Phenylethyl acetate | 6.62±0.34 | 8.44±0.63 | 18.72±0.03 | 12.24±1.82 | 18.96±1.55 | 8.95±0.07 | 21.50±1.10 |
| Ethyl laurate | 17.47±0.18 | -- | 23.59±0.09 | 13.78±1.07 | 11.33±0.43 | 7.59±0.17 | 83.78±0.42 |
| Ethyl myristate | 3.14±0.11 | -- | -- | 9.23±3.11 | -- | -- | 30.34±3.45 |
| Diethyl azelate | 1.14±0.17 | 4.02±1.07 | -- | 1.31±0.05 | -- | -- | 4.72±0.27 |
| Ethyl palmitate | 9.60±0.78 | 48.67±1.65 | 77.65±1.03 | 80.17±4.02 | 66.00±0.25 | 88.73±2.86 | 107.99±5.76 |
| Benzoyl acid 2-Chloroethyl ester | -- | -- | -- | 0.47±0.02 | -- | -- | -- |
| Acetyl tributyl citrate | 17.04±0.20 | -- | -- | 9.85±0.23 | -- | -- | -- |
| Tributyl citrate | -- | -- | -- | -- | -- | -- | 1.71±0.87 |
| Guaiacol | 1.45±0.21 | 0.29±0.01 | 0.72±0.02 | 1.42±0.11 | 1.76±0.14 | -- | 0.58±0.10 |
| 4-Vinyl guaiacol | -- | 23.27±2.69 | 18.94±1.32 | 17.06±0.13 | 16.24±1.44 | 16.17±0.45 | 42.54±11.04 |
| Phenol | 1.64±0.08 | 2.16±0.10 | -- | 0.82±0.08 | -- | 2.98±0.04 | -- |
| 4-Isopyrenyl phenol | -- | -- | -- | -- | -- | -- | 1.05±0.42 |
| Benzestrol | 1.24±0.06 | -- | -- | -- | -- | -- | -- |
| 4-Tert-butyl phenol | -- | -- | -- | 7.40±0.20 | -- | -- | -- |
| 2,5-Di-tert-butyl hydroquinone | -- | -- | -- | -- | -- | -- | 7.05±0.45 |
| 15-Crown-5 | -- | -- | -- | 10.26±0.00 | -- | -- | 11.16±0.65 |
| 1,1-Diethethoxyethane | 32.51±1.58 | 26.52±3.53 | 54.00±0.67 | 37.68±0.07 | 25.20±1.73 | 19.61±0.64 | 23.63±1.35 |
| Octamethylcy clotetrasiloxane | 1.64±0.08 | -- | -- | 2.29±0.07 | -- | -- | -- |
| Decamethylic cyclopentasiloxane | 3.78±0.18 | -- | 0.87±0.00 | 4.91±0.50 | 0.77±0.12 | 0.93±0.05 | -- |
| Dodecamethyl cyclohexasiloxane | -- | 1.83±0.91 | 1.79±0.04 | 7.92±0.21 | -- | 1.80±0.11 | 8.61±0.94 |
| Styrene | -- | -- | -- | -- | -- | -- | 4.09±0.23 |
| 1,9-Decadienene | -- | -- | -- | 2.59±0.09 | -- | -- | -- |
| Naphthalene | -- | -- | -- | 3.71±0.77 | -- | 10.72±0.30 | 2.54±0.57 |

Note: "--" indicates not detected.
